# Controlled Aloin Release from Crosslinked Polyacrylamide Hydrogels: Effects of Mesh Size, Electric Field Strength and a Conductive Polymer

**DOI:** 10.3390/ma6104787

**Published:** 2013-10-22

**Authors:** Sumonman Niamlang, Tawansorn Buranut, Amornrat Niansiri, Anuvat Sirivat

**Affiliations:** 1 Department of Materials and Metallurgical Engineering, Faculty of Engineering, Rajamangala University of Technology Thanyaburi, Klong 6, Thanyaburi, Pathumthani 12110, Thailand; E-Mails: tawansorn.b@en.rmutt.ac.th (T.B.); amornrat.n@en.rmutt.ac.th(A.N.); 2 Conductive and Electroactive Polymers Research Unit, The Petroleum and Petrochemical College, Chulalongkorn University, Bangkok 10330, Thailand; E-Mail: anuvat.s@chula.ac.th

**Keywords:** aloin, iontophoresis, transdermal drug delivery, conductive polymer

## Abstract

The aim of this paper is to investigate the effects of hydrogel mesh size, a conductive polymer, and electric field strength on controlled drug delivery phenomena using drug-loaded polyacrylamide hydrogels prepared at various crosslinking ratios both with and without a conductive polymer system. Poly(*p*-phenylene vinylene), PPV, as the model conductive polymer, was used to study its ability to control aloin released from aloin-doped poly(*p*-phenylene vinylene)/polyacrylamide hydrogel (aloin-doped PPV/PAAM). In the passive release, the diffusion of aloin from five aloin-doped PPV/PAAM hydrogel systems each was delayed ranging from during the first three hours to during the first 14 h due to the ionic interaction between the anionic drug and PPV. After the delayed periods, aloin could diffuse continuously into the buffer solution through the PAAM matrix. The amount of aloin released from the aloin-doped PPV/PAAM rose with increasing electric field strength as a result of the three mechanisms: the expansion of PPV chains inside the hydrogel, iontophoresis, and the electroporation of the matrix pore size, combined. Furthermore, the conductive polymer and the electric field could be used in combination to regulate the amount of release drug to a desired level, to control the release rate, and to switch the drug delivery on/off.

## 1. Introduction

Aloe vera has been in use for centuries because of its curative and therapeutic properties and there are extensive research studies covering at least 75 active ingredients found inside the inner gel of the plant, each of which has its own unique therapeutic effects. Extracted from aloe, Aloin, Aloe-emodin, and Aloesin are active compounds that help reduce pain and inflammation, stimulate skin growth, and repair damaged skin cells. Due to the low content of active compounds (Aloin, Aloe-emodin, Aloesin, Glycoprotien or polysaccharides (<5% v/v)) in the plant, the development of a controlled extraction system was required to increase the efficacy of therapeutic aloe vera [1]. A transdermal drug delivery system is a system that delivers drugs to the circulatory system through human skin, by which the amount of drug released into the blood can be regulated to the desired therapeutic levels [2]. Even though the transdermal route is ideal for the drug delivery, its effectiveness is weakened by the natural blocking ability of human skin, especially by the *stratum corneum* layer, the top layer of the epidermis [3]. There are however a number of techniques to circumvent this problem, such as by removing the *stratum corneum*, generating a pathway through the *stratum corneum*, or applying external stimuli [4]. Among the external stimuli, the application of an external electric field is an attractive method for regulating release drug because the electric field allows control of the amount of drug released merely by adjusting the applied voltage [5]. Widely used in the controlled drug delivery system, hydrogels are three-dimensional high molecular weight networks composed of a polymer backbone, water, and a crosslinking agent, all of which are the main elements present in the transdermal controlled drug delivery patch [5]. Recently, the requirement for a highly effective transdermal delivery system has been broadened from merely having high drug permeability through the human skin at a therapeutic level to encompassing ability to control the rate of drug delivery and to switch on/off the release profiles. Conductive polymer can offer the possibility of controllable drug delivery through electrical stimulation [6]. When an electric field is applied, the oxidation level of the conductive polymer changes via the oxidation-reduction reaction, thereby releasing the drug having loaded on the conductive polymer chain. Thus, conductive polymer is suitable for an on/off switchable controlled drug delivery system.

However, the application of conductive polymer to the controlled drug delivery contains limitations, *i.e.*, limited choices of dopants and low permeability of the high molecular weight of delivered drug, both of which affect the viability of this option [6,7]. To overcome such limitations, a composite of a drug doped with conductive polymer and hydrogel was examined. Polyacrylamide hydrogel or PAAM was chosen as the model hydrogel and poly(*p*-phenylene vinylene) or PPV as the model conductive polymer. PAAM hydrogel is an electro-responsive hydrogel, which varies its mesh size in response to the external electric field applied. As such, the amount of released drug, the rate of released drug, and the on/off switchable release profile can be easily controlled by adjusting the supplied voltage [7].

In this work, the aloin-doped PPV/PAAM hydrogel systems were prepared at various crosslinking ratios to investigate the effects of mesh size on drug delivery phenomena. The release profile and release kinetic of the aloin from the transdermal path with and without conductive polymer were examined at various electric field strengths to study the effects of the conductive polymer and electric field strength.

## 2. Methodology

### 2.1. Materials

Aloin (AR grade, Sigma-Aldrich Chemie GmbH, MO, USA) was used as the model drug. Acrylamide (AAM) (AR grade, Sigma-Aldrich Chemie GmbH, MO, USA); N,N′-methylenebisacrylamide (N,N′-MBA) (AR grade, Loba Chemie, Loba Chemie Pvt. Ltd., Mumbai, India); tetramethylenediamine (TEMED) (AR grade, Fisher Scientific, Fisher sciencetific UK limited, Leics, UK); and ammonium peroxodisulfate (AR grade, VWR, VWR International BVBA, Lueven, Belgium) were used as the monomer, crosslinker, catalyst, and initiator, respectively. Sodium acetate (AR grade, Ajax Finechem, Nuplex Industries, New South Wales, Australia) and glacial acetic acid (AR grade, Mallinckrodt Chemicals, Paris, KY, USA) were used in this study without further purification. The α,α′-dichloro-*p*-xylene and tetrahydrothiophene (THT) (AR grade, Sigma-Aldrich Chemie GmbH, Steinheim, Germany) were used to synthesize PPV. Acetone and methanol (AR grade, VWR, VWR International S.A.S, Fontenary-sous-Bios, France) were used as received.

### 2.2. Synthesis of Poly(p-phenylene vinylene)-PPV

The PPV sample was synthesized via a polyelectrolyte precursor according to Burn *et al.*’s method (1992) [8], by which 10 g of α,α′-dichloro-*p*-xylene was dissolved in 150 mL of methanol and THT (15 mL) was subsequently added to the solution. The resulting mixture was heated in a 50 °C oil bath overnight and then 250 mL of acetone was added to precipitate the salt *p*-phenylene dimethylene bis tetramethylene sulfonium chloride. The mixture was stirred in an ice bath for 0.5 h before filtration. The derived white solid salt was cleansed with acetone and dried in vacuum at room temperature until two sequential weightings provided 85% yield [8]. 1.0 g of the washed and dried salt was dissolved in 7.5 cm^3^ of methanol and cooled to 0 °C before added to 6.3 cm^3^ of aqueous sodium hydroxide (0.4 M). After 120 min, 1 cm^3^ of hydrochloric acid (0.4 M) was added to stop the reaction, thereby yielding poly [(*p*-phenylene) bis(tetrahydrothiophenechloride)]. The 14.8 cm^3^ solution was subsequently dialyzed against a water-ethanol mixture (1:1, 3 × 1000 cm^3^) for a period of three days to remove low molecular weight pre-polymer and impurities. The dialyzed poly [(*p*-phenylene) bis(tetrahydrothiophenechloride)] solution was poured onto a glass dish and allowed to evaporate at room temperature in free air stream. After 24 h, the yellowish-green precursor films were heated at 200 °C for 16 h in a vacuum oven to yield PPV film. The obtained PPV film was ground with a jar mill for 2 days.

### 2.3. Preparation of Aloin Doped Poly(p-phenylene vinylene), Aloin Doped PPV

The aloin doped PPV was prepared by acid-assisted redox doping reaction as illustrated in Equation (1):

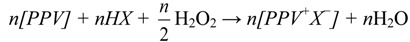
(1)
where *[PPV]* denotes a repeating unit of the conjugated PPV polymer, *HX* the functionalized aloin, and *n* the number of moles of the substances [9]. In this reaction, hydrogen peroxide (H_2_O_2_) was chosen as the oxidant reagent.

### 2.4. Electrical Conductivity Measurements

**Figure 1:**
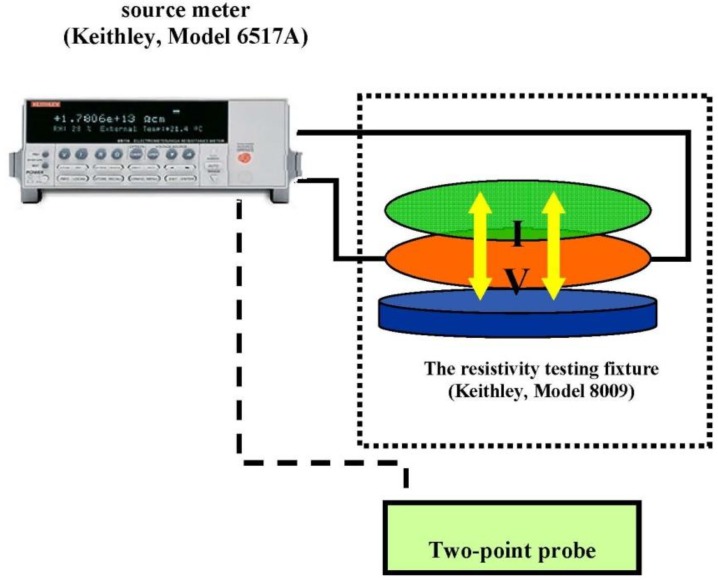
Electrical conductivity measurement.

The electrical conductivity of the PPV and the aloin doped PPV were measured using a custom-made two-point probe which was connected to a voltage supplier (Keithley, Keithley Instruments, Inc., Cleveland, OH, USA, 6517 A) in which its voltage was varied and the resultant current was measured as shown in Figure 1. The electrical conductivity was calculated using the below equation:

σ *=* (*I/KV*)·*t* (2)

where *I* signifies the measured current (A), *V* the applied voltage (V), *t* the thickness, and *K* the geometric correction factor of the two-point probe, which is determined by calibrating the probe with a silicon wafer with known resistivity value [10].

### 2.5. Preparation of Aloin-Loaded Polyacrylamide Hydrogel (Aloin/PAAM)

1.27% w/w of aloin-loaded PAAM hydrogels (based on the weight of the acrylamide monomer) was prepared by the free-radical polymerization of 2.32 g of acrylamide in an aqueous solution of aloin with N,N′-MBA as crosslinker [11]. Ammonium persulfate and TEMED were respectively used as the initiator and the accelerator. To study the effect of the crosslinking ratio on the release of the aloin from aloin-loaded PAAM hydrogels, gels at various crosslink ratios (mol MBA: mol AAM; 0.001, 0.002, 0.005, 0.010, 0.024; PAAM_01, PAAM_02, PAAM_03, PAAM_04, PAAM_05, respectively) were prepared with various amounts of N,N′-MBA.

### 2.6. Preparation of Aloin-Doped PPV/PAAM Hydrogel

The aloin-doped PPV/PAAM hydrogels were prepared by the free-radical polymerization of 2.32 g of acrylamide in an aqueous solution with 7.5 mg of aloin-doped PPV, N,N′-MBA, and ammonium persulfate and TEMED, and then cast in a mold as described in the preparation of aloin-loaded PAAM hydrogels.

### 2.7. Characterization of PAAM Hydrogel, Aloin-Loaded PAAM Hydrogel and Aloin-Doped PPV/PAAM Hydrogel

To investigate the morphology of the PAAM hydrogels at various crosslink ratios with and without an electric field, scanning electron micrographs of the hydrogels were taken using an acceleration voltage of 15 kV and a magnification of 350. Samples were prepared from frozen swollen hydrogels with and without electric field in liquid nitrogen and then dried in vacuum at −50 °C.

To determine the % swelling in buffer solution of the PAAM hydrogels at various crosslink ratios, the hydrogels were immersed in an acetate buffer, pH 5.5, at 37 °C. After 5 days, the swollen PAAM hydrogels were removed, gently wiped to remove the surface water, and then weighed.

To determine the % swollen PAAM hydrogels were dried in a vacuum oven for 5 days until constant weight values were attained. The % swelling was calculated using the following equation [12]:

(3) $$\text{Degree of swelling }\left(\%\right)\;=\frac{M-M_{d}}{M_{d}}\times 100$$

where *M* denotes the weight of the swollen sample, and *M_d_* is the weight of the swollen sample after drying in a vacuum oven [12]. All reported data were average values taken from repeated measurements using five specimens. The hydrogel mesh size *ξ* was calculated using the following equation:

(4) $$\xi =v_{2,s}{\left[C_{n}\left(\frac{2M_{c}}{M_{r}}\right)\right]}^{1/2}l$$

where *C_n_* is the Flory characteristic ratio for PAAM (8.8), and *l* is the carbon-carbon bond length (=15.4 Å) [13].

The value of molecular weight between crosslinks, *M_c_*, was calculated from the swelling data as in Equation (5) [13]:

(5) $$\frac{1}{{\bar{M}}_{c}}=\frac{2}{{\bar{M}}_{n}}-\frac{\bar{\upsilon }/v_{1}[\ln(1-\upsilon_{2,S})+\upsilon_{2,S}+{\chi \upsilon_{2,S}}^{2}]}{\upsilon_{2,r}[{\left(\upsilon_{2,s}/\upsilon_{2,r}\right)}^{1/3}-1/2(\upsilon_{2,s}/\upsilon_{2,r})]}$$

where *M_n_* denotes the number-average molecular weight of the polymer before crosslinking (36,400 g/mol), *υ* the specific volume of PAAM (0.741 cm^3^/g), *v*_1_ the molar volume of the water (18.1 cm^3^/mol), *υ*_2,*r*_ the volume fraction of the polymer in the relaxed state, *υ*_2,*S*_ the volume fraction of the polymer in the swollen state, and *χ* the Flory polymer-solvent interaction parameter for PAAM/water (0.48) [13].

The Differential Scanning Calorimetry (DSC) thermograms of the aloin, PAAM hydrogel, the aloin-loaded PAAM hydrogel, and aloin-doped PPV/PAAM samples were recorded to determine their thermal behavior. To study DSC thermograms, 2 to 4 mg of each sample was accurately weighed in an aluminum pan with a sealed cover. The measurements were performed under N_2_ atmosphere over 30 to 400 °C at heating rate of 10 °C/min.

The absorption infrared spectra of the pristine PPV and aloin-doped PPV were measured with an attenuated total reflection Fourier transform infrared spectrometer (ATR-FTIR; Thermo Nicolet, Nexus 670) to confirm the doping of aloin on the PPV chain. The samples were placed on a zinc selenide (ZnSe) crystal sample holder.

### 2.8. Drug Release Studies

Diffusion through pig skin was carried out in order to study the release characteristics of the aloin from the aloin-loaded PAAM and the aloin-doped PPV/PAAM hydrogels. A piece of pig skin was placed on top of a custom-built modified Franz diffusion cell filled with acetate buffer solution [14]. The pig skin was allowed to contact with the acetate buffer (pH 5.5; the normal human skin acidity) in the receptor chamber until reaching equilibrium; the buffer solution was magnetically stirred throughout the experiment period (48 h) at a thermostatically maintained temperature (37 ± 2 °C). The aloin-doped PPV/PAAM hydrogels with specific crosslinking ratios (mol MBA: mol AAM = 0.002, 0.005, 0.016, and 0.024 for PAAM_1, PAAM_2, PAAM_3 and PAAM_4, respectively) were placed between a copper cathode and the net, which was mounted onto the receptor compartment. To study the effect of electric field strength on the release of the aloin from the aloin-loaded PAAM and the aloin-doped PPV/PAAM hydrogels, the cathode electrode (copper electrode) was connected to a power supply, which provided various electrical voltages across the hydrogel, pig skin, and the buffer solution. The anode electrode pin was positioned in the buffer solution. The amount of drug in the withdrawn solution sample was determined using a UV spectrophotometer (259 nm). The experiments were carried out in triplicate and the data were reported as average values.

## 3. Results and Discussion

### 3.1. Characterization PAAM Hydrogel, Aloin-Loaded PAAM Hydrogel and Aloin-Doped PPV/PAAM Hydrogel

**Figure 2:**
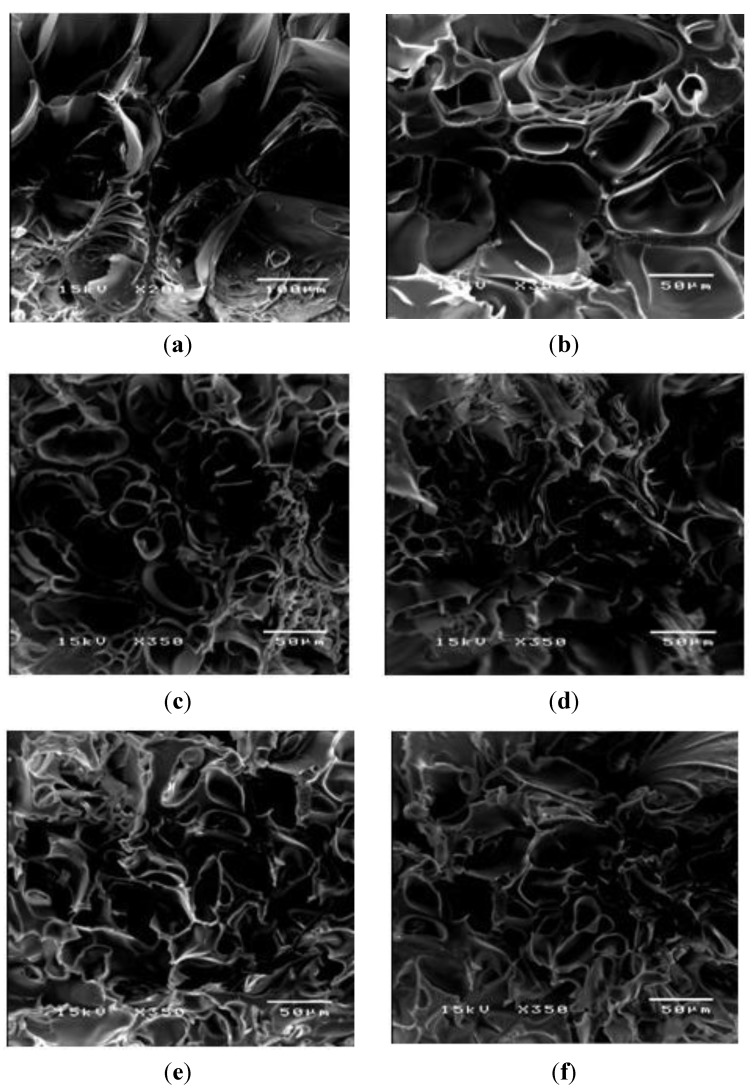
The morphology of PAAM hydrogel after swelling: (**a**) PAAM_1; (**b**) PAAM_2; (**c**) PAAM_3; (**d**) PAAM_4; (**e**) PAAM_5; and (**f**) PAAM_6 at magnification of 350.

**Figure 3:**
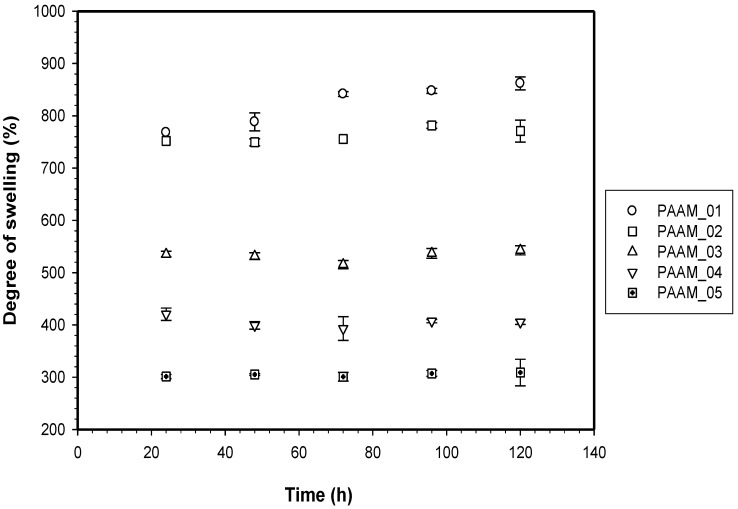
Degree of swelling of polyacrylamide hydrogel.

PAAM was polymerized through free radicalization and subsequently crosslinked at 27 °C [11]. The calculated mesh sizes of PAAM hydrogel were PAAM_01, 292 ± 8 Å; PAAM_02, 183 ±16 Å; PAAM_03, 161 ± 8 Å; PAAM_04, 148 ± 3 Å; PAAM_05, 99 ± 2 Å. As the crosslinking ratio decreases, the mesh size increases. Scanning electron micrographs of PAAM hydrogels at various crosslinking ratios are shown in Figure 2. The bulk hydrogel mesh sizes also decreased with increasing crosslinking ratio. As the amount of crosslinking agent decreased, the spacing between the crosslinks became wider. The apparent mesh sizes, determined visually from the SEM micrographs, were greater than the mesh sizes calculated from Equation (4). The calculated mesh sizes are bulk mesh size calculated from every part of the gel, while mesh sizes from SEM micrographs are the apparent mesh size at the outer surface. Nonetheless, the results from both methodologies explicitly indicated that the crosslinking ratio decreased as the mesh size increased [15]. In this work, the degree of swelling was related to the amount of gel required to achieve a suitable degree of swelling for transdermal drug delivery patch. As intuitively expected, the degree of swelling was inversely proportional to the degree of crosslinking as shown in Figure 3. The degree of swelling of the five crosslinked PAAMs almost reached the equilibrium values of 300%–800% after the 20th hour. These results are consistent with theoretical predictions, which describe the swelling of gel as a function of the degree of crosslinking [15].

DSC thermograms of aloin-loaded PAAM hydrogel and PAAM hydrogel were measured to investigate the interaction between aloin and the polyacrylamide matrix. The melting temperature (*T*_m_) of PAAM was 219 °C [16]. However, the *T*_m_ of PAAM in aloin-loaded PAAM was 241 °C, suggesting that aloin possibly interacted with the PAAM hydrogel through hydrogen bonding between the hydroxyl groups of the aloin and the amine groups of the PAAM hydrogel.

**Figure 4:**
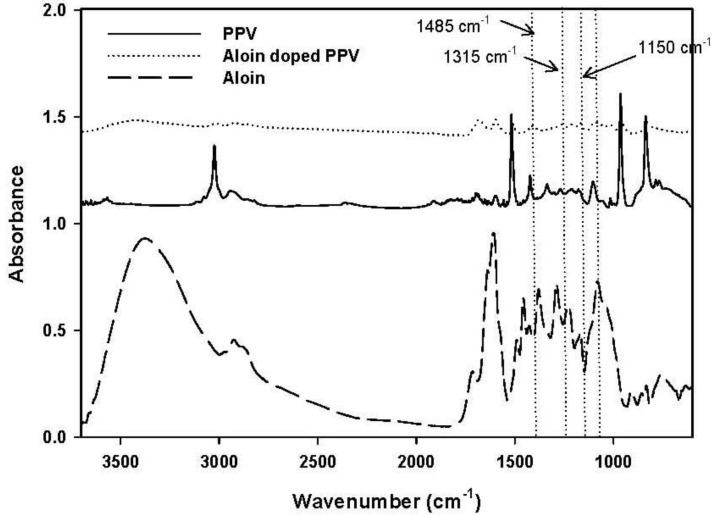
Absorption infrared spectra of poly(phenylene vinylene), PPV; aloin; and aloin doped poly(phenylene vinylene), aloin doped PPV.

To confirm the success of doping aloin on PPV chain, ATR-FTIR was used. FTIR spectra of synthesized PPV, aloin, and aloin-doped PPV are shown in Figure 4. The adsorption peaks of the pristine PPV were 3022, 550, 830 and 1511 cm^−1^, each of which respectively represented the trans vinylene C–H stretching mode, the phenylene out of plane ring bending, the *p*-phenylene ring C–H out of plane bending, and the C–C ring stretching [17]. The FTIR spectrum of aloin-doped PPV showed new bands at 1485, 1315 and 1150 cm^−1^. The emergence of these new bands in the spectra was due to the formation of the quinoid structure, which was the result of symmetric breaking of the polymeric chain. Although the formation of the quinoid structure occurred in the presence of the doping agent, certain FTIR peaks (3022, 550, 830 and 1511 cm^−1^) still remained after the doping process. Therefore, even after the extensive oxidation process, only partial oxidation of the polymer took place and the two structures, *i.e.*, quinoid and benzoid structures, coexisted.

Before doping aloin on the PPV chain, the PPV film was ground into irregular shape powder (diameter <68 μm). The electrical conductivity of the pristine PPV and that of aloin-doped PPV were measured, the former of which was 7.89 × 10^−8^ ± 1.05 × 10^−9^ S/cm and the latter 5.28 × 10^−7^ ± 1.72 × 10^−8^ S/cm. After the doping process, ions were created on the conductive polymer backbone in which the higher the ion mobility, the better the electrical conductivity. Better conductivity is a good indicator that aloin is successfully doped on PPV chains.

### 3.2. Release Characteristics

**Figure 5:**
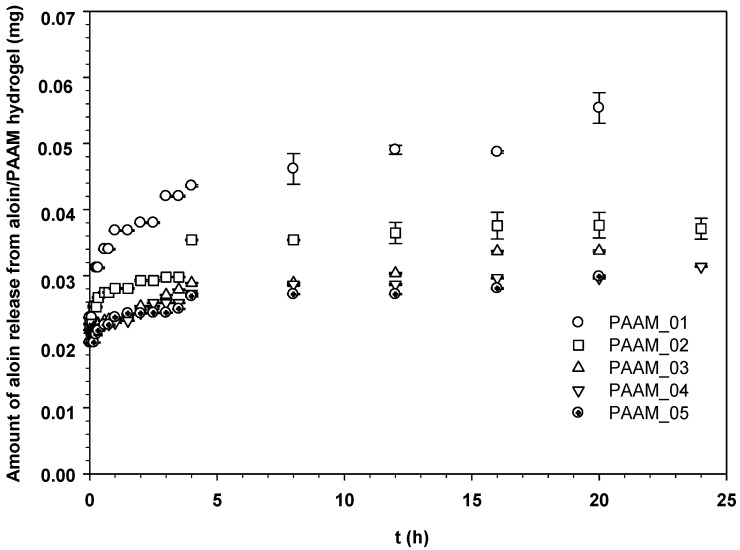
Amounts of aloin released from aloin/PAAM hydrogel at time *t vs. t* (h) at various crosslinking ratios, E = 0 V, pH = 5.5, and at 37 °C.

The amount of aloin released through the pig skin was reported as the amount of aloin released from aloin-loaded PAAM as shown in Figure 5. In the passive release characteristic (E = 0 V), the amounts of aloin released from aloin-loaded PAAM were noticeably high during the first 3 h and reached the equilibrium value afterward. Evidently, the amount of aloin released from aloin-loaded PAAM through the pig skin was greater at a given time for samples with a lower crosslinking ratio [17]. A lower crosslinking ratio represents a larger hydrogel mesh size, suggesting that the deliver pathway is larger and thereby a greater quantity of released drug is obtained [18].

**Figure 6:**
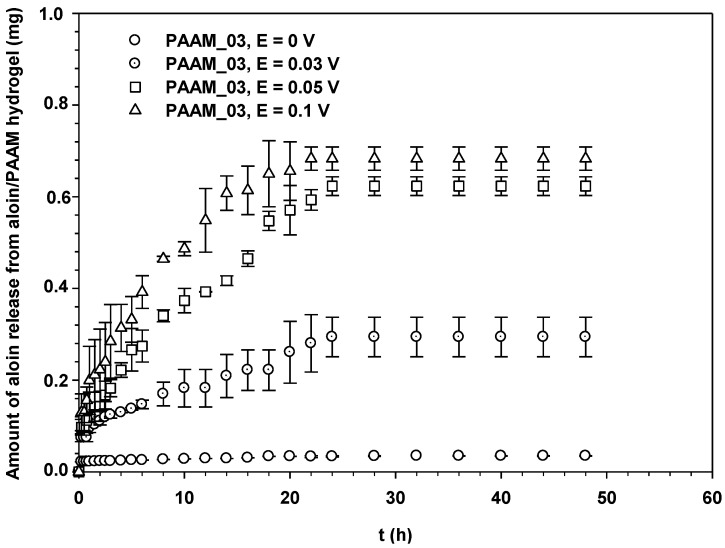
Amounts of aloin released from aloin/PAAM hydrogel (PAAM_03) at time *t vs. t* (h) at various electric field strengths, pH = 5.5, and at 37 °C.

Figure 6 shows the amounts of aloin released from aloin-loaded PAAM in relation to time at various electric field strengths, 0–0.1 V. Each sample was attached to the negatively charged electrode (cathode). From Figure 5, it is evident that the amount of aloin released from aloin/PAAM was greater at a higher electric field strength due to three driving forces: electrostatic force, the modified pathway of pig skin, and expansion of PAAM hydrogel. As the electric field was applied, the electrons pushed the anionic out and generated small pathways in the pig skin. Thus, the higher the electric field strength, the greater the amount of aloin released. The third driving force, *i.e.*, expansion of PAAM hydrogel, was the direct result of the expansion of the PAAM hydrogel pore size following the application of the electric field [5,19].

**Figure 7:**
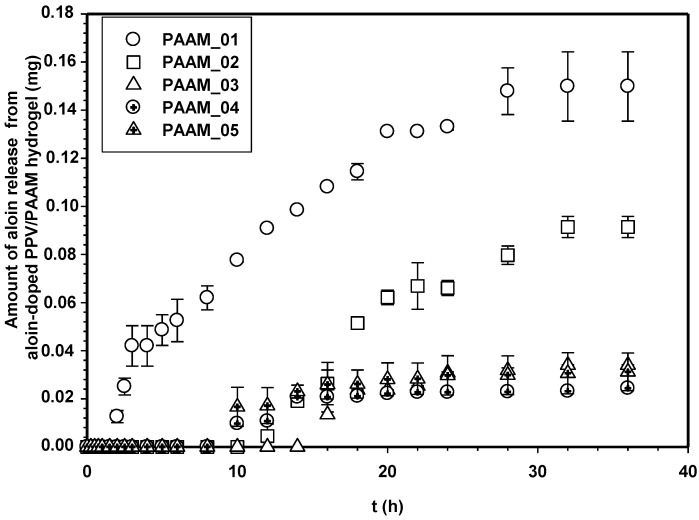
Amounts of aloin released from aloin-doped PPV/PAAM hydrogel at various crosslinking ratios, E = 0 V, pH = 5.5, and at 37 °C.

**Figure 8:**
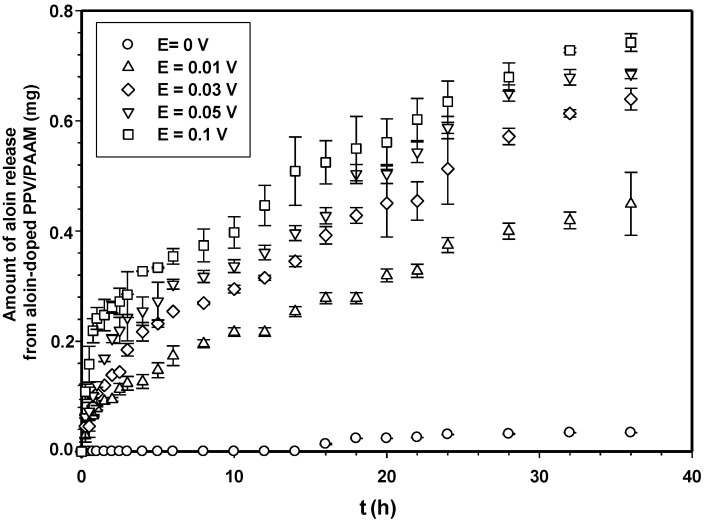
Amounts of aloin released from aloin-doped PPV/PAAM (PAAM_03) hydrogel at various crosslinking ratios, E = 0 V, pH 5.5, and at 37 °C.

Figure 7 shows the amounts of aloin released from aloin-doped PPV/PAAM vis-à-vis time at electric field strength 0 V. In the absence of an electric field, no aloin molecules were released from the aloin-doped PPV/PAAM during the first 3, 10, 14, 8 and 8 h for PAAM_01, PAAM_02, PAAM_03, PAAM_04 and PAAM_05, respectively. After their respective no-release periods, the amounts released gradually increased until reaching their equilibriums. The amounts of aloin released also increased with increasing crosslinking ratios after no-release periods [7,19,20]. The amount of aloin released from aloin-doped PPV/PAAM (PAAM_03) was selected to study the release characteristic under applied electric field. Figure 8 shows the release profile of aloin-doped PPV/PAAM (PAAM_03) at various electric field strengths, 0–0.1 V. The amount of aloin released from aloin/PAAM was greater at a higher electric field strength due to the stronger reduction reaction of aloin-doped PPV. When an electric field was applied, PPV, conductive polymer, was reduced and PPV chains expanded, creating a larger free volume in the hydrogel and thereby facilitating the diffusion of aloin through the PAAM matrix [7,19,20,21]. To describe the release mechanisms of aloin from aloin-doped PPV/PAAM hydrogel system, four combinations of driving forces were used, the four of which were the expansion of conductive polymer chain, the electrostatic force between electron and drug, the modified pathway of pig skin, and expansion of PAAM hydrogel. As the external electric field was applied, the conductive polymer was reduced, conductive polymer chains were expanded, and the free space in hydrogel matrix was generated, thus electric field pushing the ionic drug out by electrostatic force. Moreover, the electric field created the micro pathway in the pig skin while simultaneously expanding the hydrogel mesh size. As a result, the amount and the rate of drug released increase with the application of external electric field.

As reported in our previous work, the diffusion coefficients and the release mechanisms of salicylic acid-doped poly(phenylene vinylene)/polyacrylamide hydrogels (SA-doped PPV/PAAM) were investigated. The results show the similar release mechanism. In the absence of an electric field, the diffusion of SA from the SA-doped PPV/PAAM is delayed in the first 3 h due to the ionic interaction between the anionic drug (SA anion) and the PPV. Beyond this period, SA is dissolved in and can diffuse into the buffer solution through the PAAM matrix [20].

### 3.3. Drug Release Kinetics

The effects of the electric field strength on the release kinetic of aloin from aloin-loaded PAAM hydrogel were subsequently investigated. There are several models that can interpret the controlled release behavior [22], one of which is the Higuchi’s square-root equation, which is ideal for the release from a polymeric matrix (insoluble in the solvent) at a “pseudo steady-state”.

(6) $$\frac{M_{t}}{M_{\infty }}=k_{H}t^{1/2}$$

where *M_t_* denotes the amount of drug release at time *t*, *M_∞_* the amount of drug release at infinity, *k_H_* the Higuchi’s kinetic constant, and *t* the release time. The diffusion coefficients of aloin from aloin-loaded PAAM hydrogel could be calculated from the slopes that were obtained from the plots of drug mass accumulation against square root of time (data from Figure 4 and Figure 5) according to the Higuchi’s Equation [21]:

(7) $$Q=2C_{o}{\left(\frac{Dt}{\pi }\right)}^{1/2}$$

where *Q* denotes the amount of drug mass released per unit cross-section of the barrier at time *t*, *C*_o_ the initial drug concentration in the hydrogel, and *D* the diffusion coefficient of the drug.

**Figure 9:**
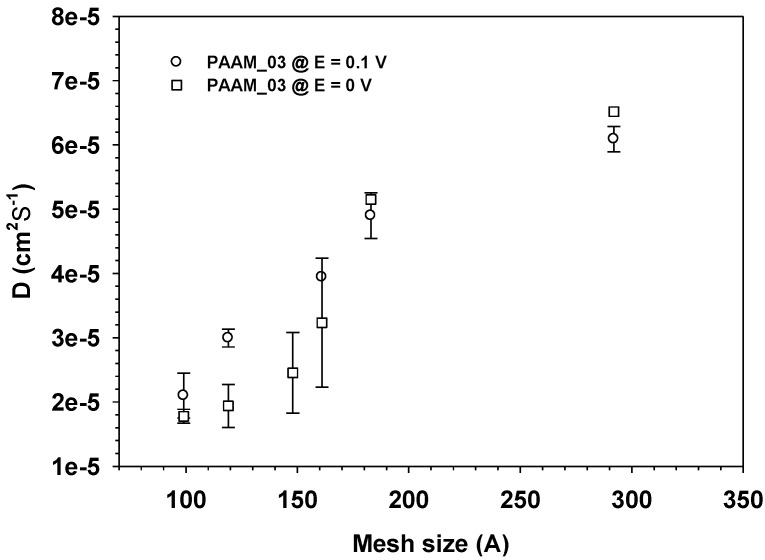
The diffusion coefficient, D_app_ of Aloin from Aloin/ PAAM hydrogel (E = 0 and 0.1 V) *vs.* Mesh size at pH = 5.5, and at 37 °C.

*D* of aloin from aloin-loaed PAAM increased monotonically with increasing mesh size and electric field as shown in Figure 9. In general, we may conclude that the diffusion coefficient of a drug in a transdermal delivery system depends upon drug size and electric field (driving forces).

## 4. Conclusions

To increase the efficacy of aloe vera extract, the aloin-loaded PAAM and aloin-doped PPV/PAAM were prepared to use as the transdermal drug delivery patch. The aloin-loaded PAAM at various crosslinking ratios were prepared to study the effects of pore size on the release profile both with and without applying electric field strengths (0–0.1 V). The amount of released aloin and diffusion coefficient, *D*, increased with increasing hydrogel mesh size and electric field strength. As the electric field was applied (0–0.1 V), the amount of released aloin increased with increasing electric field strength which drove ionic drug through polyacrylamide hydrogel with increase in electrostatic force, modified the pathway of pig skin, and expanded PAAM hydrogel pore size. For drug doped conductive/hydrogel system, in the absence of an electric field (passive release) the diffusion of aloin from the aloin-doped PPV/PAAM hydrogel was delayed in the first 3–14 h due to the ionic interaction between the anionic drug and PPV. After 14 h, aloin could continuously diffuse into the buffer solution through the PAAM matrix. The amount of aloin released from aloin-doped PPV/PAAM rose with increasing electric field strength because of the combination of these mechanisms: the expansion of PPV chains inside the hydrogel which was the result of the reduction reaction under the negative pole, thus driving the aloin through the PAAM matrix; iontophoresis; and the electroporation of the matrix pore. Hence, the amount of drug released, the rate of drug released, and the switchable release profile can easily be controlled by adjusting the supplied voltage in the conductive/hydrogel systems.
